# A HEART-WISE: Allogeneic Wharton’s Jelly-derived Mesenchymal Stromal Cells’ Intracoronary Transplantation in Pediatric Patients with Dilated Cardiomyopathy: The First Case Reports

**DOI:** 10.1186/s12872-026-05866-x

**Published:** 2026-04-27

**Authors:** Hoda Madani, Sara Pahlavan, Massoud Vosough, Maryam Barekat, Kiara Rezaei-kalantari, Nasser Aghdami, Ali Akbar Zeinaloo

**Affiliations:** 1 Department of Applied Cell Sciences, Faculty of Basic Sciences and Advanced Technologies in Medicine, Royan Institute, ACECR, Tehran, Iran; 2 Department of Regenerative Medicine, Cell Science Research Center, Royan Institute for Stem Cell Biology and Technology, ACECR, Tehran, Iran; 3 Department of Stem Cells and Developmental Biology, Cell Science Research Center, Royan Institute for Stem Cell Biology and Technology, ACECR, Tehran, Iran; 4 Department of Radiology, Medical and Research Center, Cardio-Oncology Research Center, Rajaie Cardiovascular, Iran University of Medical Sciences, Tehran, Iran; 5 Department of Pediatrics, Pediatric Center of Excellence, Children’s Medical Center, Tehran University of Medical Sciences, Tehran, Iran; 6 Fetal & Pediatric Cardiovascular Research Center, Children’s Medical Center, Tehran University of Medical Sciences, Tehran, Iran

**Keywords:** Pediatric Dilated Cardiomyopathy, PDCM, Wharton’s Jelly-Derived Mesenchymal Stromal Cells, GMP-compliant MSC, Intracoronary Cell Transplantation, Regenerative Medicine

## Abstract

**Background:**

Pediatric dilated cardiomyopathy (PDCM) is a severe cardiovascular disorder characterized by ventricular systolic dysfunction, with heart transplantation as the gold standard therapy. Mesenchymal stromal cells (MSCs) have demonstrated promise for treating cardiomyopathy, but previous studies in pediatric patients have focused exclusively on autologous cells such as MSCs, while allogeneic MSCs with better results were studied only in adults.

**Objective:**

The study “A HEART-WISE” is the first case reports to evaluate the safety and feasibility of intracoronary transplantation of allogeneic GMP-complierant Wharton’s jelly-derived MSCs (WJ-MSCs) in PDCM patients.

**Methods:**

Two pediatric patients with PDCM received a single intracoronary infusion of 1.5 × 10⁶ cells/kg WJ-MSCs. Clinical evaluations included NYHA functional class, six-minute walk test (6MWT), N Terminal pro-brain natriuretic peptide (NT-proBNP), and cardiac imaging over a 12-month follow-up period.

**Results:**

Both patients tolerated intracoronary WJ-MSC administration without serious adverse events related to the procedure. Patient 1 demonstrated marked improvements by 3 months (NYHA III to II, LVEF 33% to 48%, 6MWT 300 to 373 m, NT-proBNP 3502 to 1734 pg/mL) but passed away at 6 months follow-up due to a viral infection. Patient 2 had a persistently reduced LVEF in the 43–46% range for more than three years prior to therapy but achieved sustained improvement by 12 months (NYHA III to II, LVEF 43% to 64%, 6MWT 265 to 380 m).

**Conclusion:**

Our preliminary findings suggest the safety and feasibility of intracoronary transplantation of allogeneic WJ-MSCs in PDCM, with potential off-the-shelf therapeutic benefit. Validation of these observations in larger randomized controlled trials is needed to better evaluate long-term safety and efficacy.

**Supplementary Information:**

The online version contains supplementary material available at 10.1186/s12872-026-05866-x.

## Introduction

Pediatric dilated cardiomyopathy (PDCM) is the most common form of childhood cardiomyopathy and is characterized by systolic dysfunction due to uni- or biventricular dilation [1]. Current therapies aim to manage symptoms, slow disease progression, and prevent arrhythmias. However, heart transplantation remains the gold standard for severe cases despite challenges, including donor limitations and long-term rejection risks [1].

Stem cell therapy, particularly with mesenchymal stromal cells (MSCs) from different sources, has emerged as a promising therapeutic approach [2–4]. MSCs derived from Wharton’s jelly (WJ-MSCs) are advantageous due to their superior proliferative potential, immunomodulatory effects, and regenerative capacity while avoiding ethical concerns associated with other sources [5, 6].

While previous studies have demonstrated the safety and efficacy of allogeneic MSCs in adult non-ischemic dilated cardiomyopathy (NIDCM) [7–10], no clinical trials have explored the application of allogeneic WJ-MSCs in PDCM. This study represents the first case reports of intracoronary WJ-MSC transplantation in PDCM patients, potentially offering a novel off-the-shelf therapeutic option that may reduce the dependency on heart transplantation.

## Materials and methods

### Study design

The “A HEART-WISE” study was designed as the first case reports to evaluate the safety and feasibility of intracoronary transplantation of allogeneic WJ-MSCs in pediatric patients with DCM. Secondary endpoints included improvements in New York Heart Association (NYHA) functional classification, New York University Pediatric Heart Failure Index (NYU PHFI), six-minute walk test (6MWT), echocardiography, cardiac magnetic resonance (CMR) imaging, and laboratory-based safety markers. Ethical approval was obtained (ID: IR.TUMS.MEDICINE.REC.1399.896), and the study was registered (IRCT20201217049743N1), adhering to the principles of the “Declaration of Helsinki.”

Two pediatric patients with PDCM were selected between September 2023 and March 2024 from the Children’s Medical Center affiliated with TUMS. Patients were screened according to the inclusion and exclusion criteria outlined in Table 1. Following signed parental consent, clinical evaluations included NYHA and NYU PHFI assessments, 6MWT, echocardiographic and CMR imaging, and lab tests, particularly NT-pro brain natriuretic peptide (NT-proBNP) at baseline and follow-ups at 1 week, 1, 3, 6, 9, and 12 months. Detailed CMR-based analyses of cardiac indices were conducted at critical follow-ups of 6 and 12 months. (CRF; Supplementary File 1).

**Table 1:** Eligibility criteria

Inclusion criteria	Exclusion criteria
• Age: 4–18 y	• Secondary causes for reduced EF*
• Both genders	• Cardiogenic/ toxic shock
• Chronic heart failure disease & progressive symptoms (more than 6 months)	• Active infectious disease/ Positive viral markers (HIV, HBV, HCV, …)
• No response to standard therapy (more than 3 months)	• Active malignancy
• NYHA function class II/III	• Immunodeficiency diseases
• 20 < LVEF % <45 (echocardiography)	• Arrhythmia
• Confirmed DCM	• Coagulopathy
• Informed consent	• Uncontrolled underlying disease ◦ LFT ≥ 3 ULN ◦ Cr > 2 mg/dl

*NYHA* New York Heart Association Functional Classification, *NHU PHFI* New York University Pediatric Heart Failure Index, *LVEF* Left Ventricular Ejection Fraction, *DCM* Dilated Cardiomyopathy, *LFT* Liver Function Tests, *ULN* Upper limits of Normal

* Including critical valvular disease, severe coarctation, coronary artery anomalies, metabolic disorders, neuromuscular disorders, congenital cardiac disorders except Mitral Valve Prolapse (MVP)

### Wharton’s Jelly-derived Mesenchymal Stromal Cells (WJ-MSCs)

Allogeneic WJ-MSCs were prepared based on established protocols [11]. WJ-MSCs were isolated from umbilical cord tissue received from full-term male neonates after written informed consent was obtained for the use of their biological material. The procedure involved washing umbilical cords in phosphate-buffered saline (PBS), cutting them into 2–3 mm fragments, and enzymatic digestion using a collagenase and hyaluronidase cocktail. Following digestion, the extracted cells were cultured and harvested at passage #4. Cell expansion was performed using platelet lysate as a xeno-free culture medium.

Cell counting, cell viability, sterility, mycoplasma testing, Limulus amebocyte lysate (LAL) endotoxin assay, karyotype analysis, and flow-cytometric evaluation of positive and negative surface markers were performed in accordance with the International Society for Cell & Gene Therapy (ISCT) minimal criteria for mesenchymal stromal cells [12]. Flow-cytometric immunophenotyping was performed at the level of the working cell bank (passage 3) to confirm ≥ 95% expression of CD73, CD90, CD105, and CD29, and ≤ 2% expression of CD45, CD34, CD31, CD11b, and HLA-DR.

Accordingly, the flow-cytometry data were the same for both patients because immunophenotyping was performed at the working cell bank level, whereas all other quality-control assessments were performed separately for each patient (Supplementary Fig. 1).

The cryopreserved GMP-certified WJ-MSC product (“Whartocell^®^”, Cell Tech Pharmed Co.) was thawed, washed to remove dimethyl sulfoxide, and resuspended in 5 ml normal saline containing 2% human serum albumin. A dose of 1.5 × 10⁶ cells/kg was administered to each patient. Detailed quality control results are provided in Supplementary Table 1.

The selected dose in the present study was informed by available evidence from MSC-based cardiac cell therapy studies in adults. Despite heterogeneity in MSC source (bone marrow or umbilical cord), autologous versus allogeneic products, routes of delivery (intracoronary, transendocardial, or intravenous), and underlying pathology (ischemic vs. non-ischemic), adult trials have generally administered total cell doses ranging between approximately 20 and 100 million MSCs per patient [13–17]. To achieve a comparable total dose range while accounting for the lower body weights of pediatric patients, a body-weight–adjusted dose of 1.5 × 10⁶ MSCs/kg was selected.

### Intracoronary delivery for cell transplantation

As previously described [18], a dual-catheter technique was used for intracoronary delivery of WJ-MSCs. A conventional arterial stop-flow technique with transient coronary artery balloon occlusion was not employed. Instead, myocardial cell retention was enhanced by temporary venous outflow occlusion, achieved by transient inflation of a Swan–Ganz catheter in the coronary sinus during each injection. A second catheter was used to deliver the cells into the left and right coronary arteries (2.5 mL per injection over 30–60 s). All procedures were performed under pediatric anesthesia with continuous electrocardiographic monitoring, and patients were subsequently transferred to the pediatric cardiac intensive care unit (C-ICU) for post-procedural observation.

### Echocardiographic and cardiac magnetic resonance assessments

Transthoracic echocardiography was performed using a Philips Affiniti 70 C system with a phased‑array transducer. Standard parasternal long‑axis, short‑axis, and apical two‑ and four‑chamber views were obtained. Left ventricular internal diameters, including left ventricular end‑diastolic diameter (LVEDD) and left ventricular end‑systolic diameter (LVESD), were measured in accordance with the recommendations of the American Society of Echocardiography. Left ventricular ejection fraction (LVEF) was calculated using the biplane Simpson method by tracing the endocardial borders in end‑diastolic and end‑systolic frames. Tricuspid annular plane systolic excursion (TAPSE) was measured using M‑mode from the apical four‑chamber view. The left ventricular Tei index (Tei myocardial performance index or MPI) was assessed using Doppler echocardiography according to standard methods.

CMR imaging was performed on a 1.5 Tesla scanner (Siemens Magnetom Sola, Erlangen, Germany) using cine steady state free precession (SSFP), T1-weighted and T2-weighted myocardial mapping, and late gadolinium-enhanced sequences in pediatric DCM. A contiguous short axis stack covering the entire left ventricle from base to apex, along with standard long axis views, was acquired. Endocardial contours were manually traced at end diastole and end systole to calculate left and right ventricular end diastolic volumes (LVEDV and RVEDV), end systolic volumes (LVESV and RVESV), and ejection fractions (LVEF and RVEF) using standard volumetric analysis.

In both patients, transthoracic echocardiography included functional and dimensional parameters (including LVEF, TAPSE, LV Tei Index, and LVIDd), whereas volumetric measurements (LVEDV, LVESV, RVEDV, and RVESV) as well as ventricular ejection fractions (LVEF and RVEF) were also derived from CMR.

## Results

### Patient 1-demographic data

Patient 1 was a 13-year-old female weighing 47 kg. The disease was diagnosed at 8 years of age. Drug history included ARB (losartan 25 mg once daily), aldosterone antagonists (spironolactone 25 mg twice daily), diuretics (furosemide 20 mg once daily), and digoxin (Lanoxin 125 µg twice daily). She was a heart transplant candidate. She had a medical history of Ewing’s sarcoma at the age of 6 years and probable Doxorubicin-induced cardiomyopathy, consequently, at 8 years of age. The baseline NYHA functional class was II/III. She had a mild ST-T change on ECG. After obtaining informed consent from her parents, she underwent initial evaluations. The baseline LVEF was measured 33% on echocardiography and 22% on CMR. Analysis of the blood test for NT-proBNP showed 3502 pg/mL.

### Patient 1-efficacy outcomes (6MWT, NYHA, lab tests, echocardiography & CMR)

**Fig. 1:**
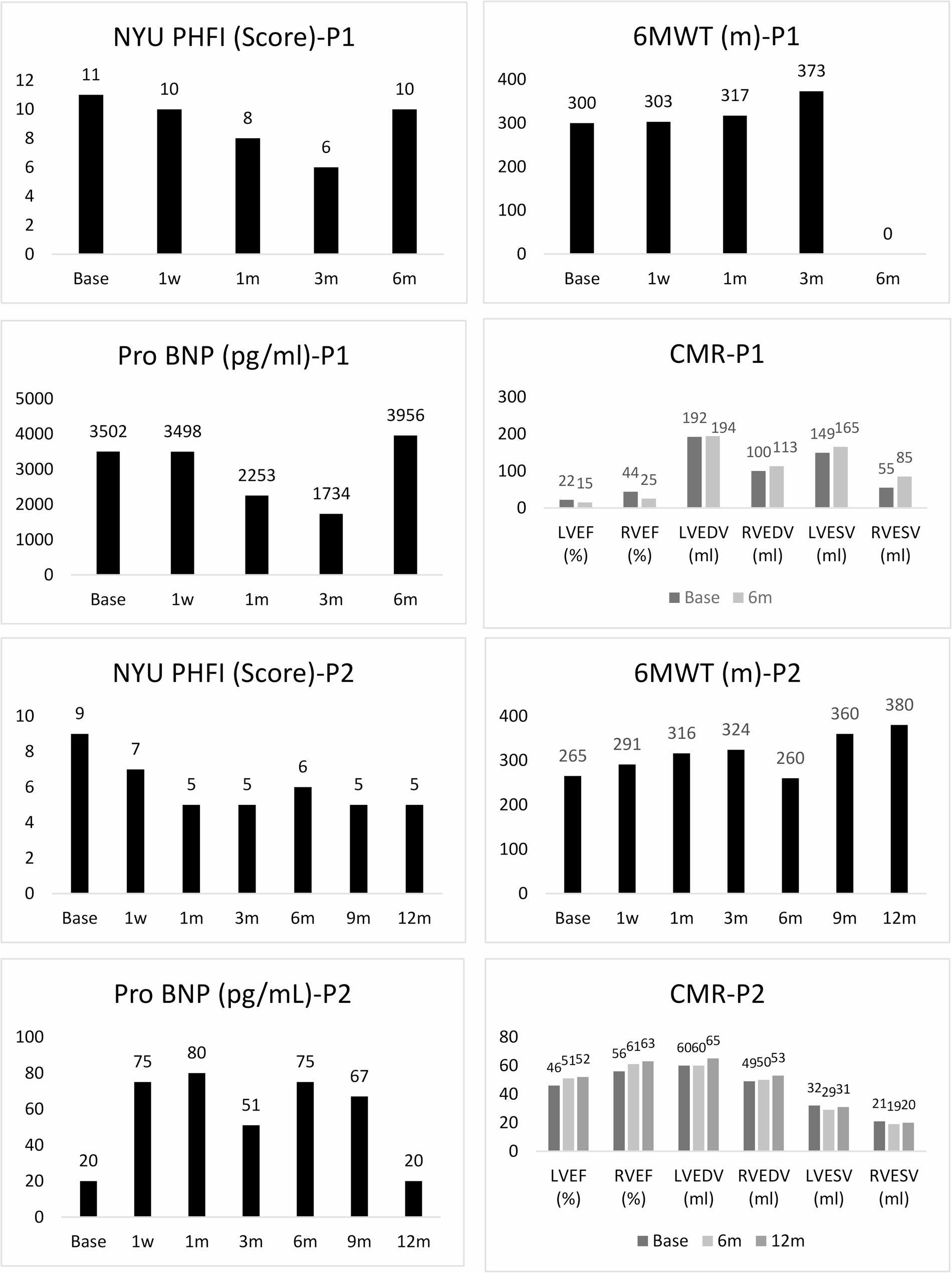
Main clinical and imaging findings including NYU PHFI, 6MWT, proBNP, and CMR results for patient 1 and 2 undergoing intracoronary transplantation of allogeneic WJ-MSCs at baseline and follow-ups. NHU PHFI: New York University Pediatric Heart Failure Index, 6MWT: Six-Minute Walk Test, Pro BNP: Pro Brain Natriuretic Peptide, CMR: Cardiac Magnetic Resonance, PDCM: Pediatric patient with DCM, LVEF: Left Ventricular Ejection Fraction, RVEF: Right Ventricular Ejection Fraction, LVEDV: Left Ventricular End Diastolic Volume, RVEDV: Right Ventricular End Diastolic Volume, LVESV: Left Ventricular End Systolic Volume, RVESV: Right Ventricular End Systolic Volume

**Fig. 2:**
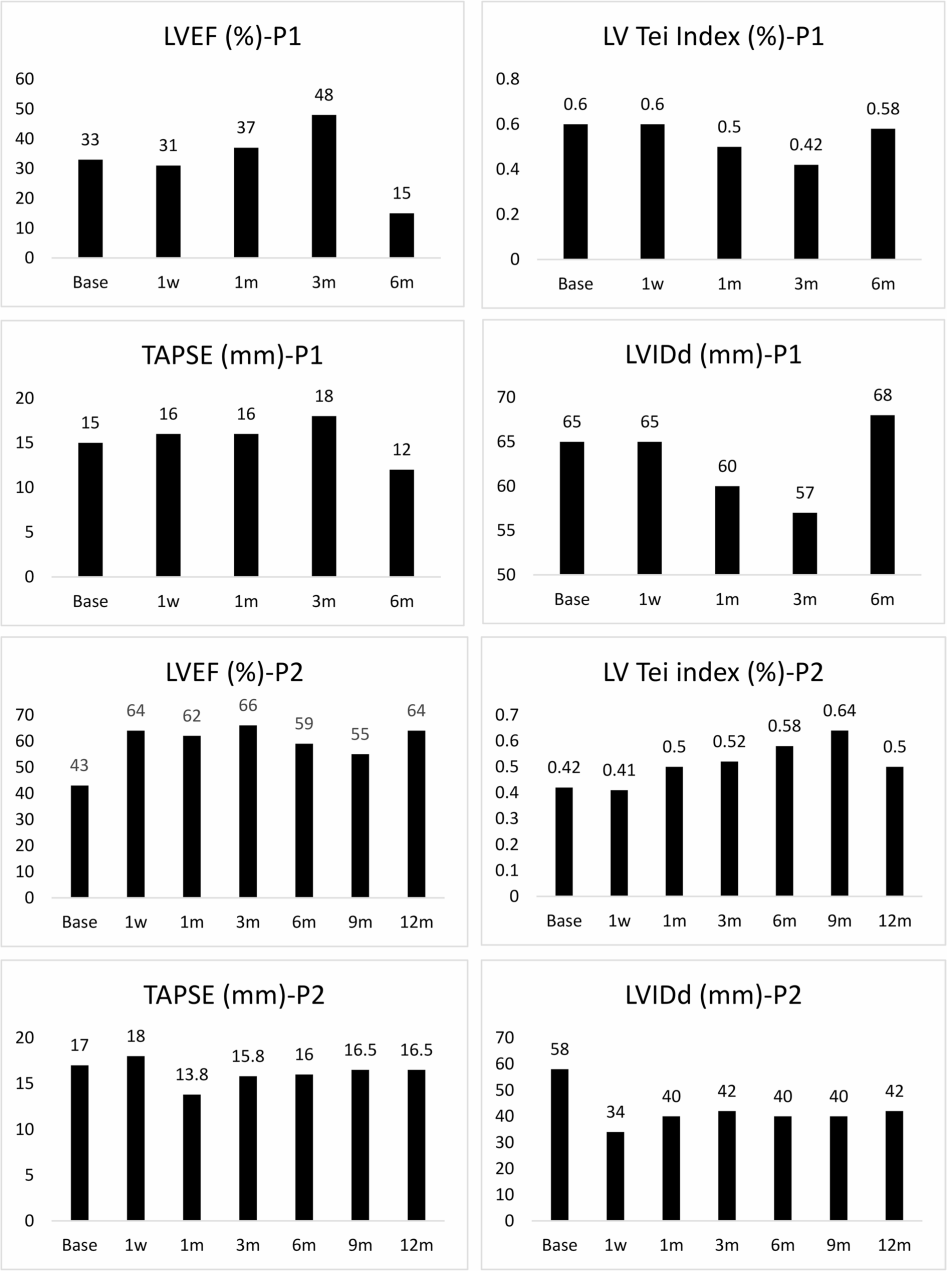
Main echocardiographic findings for patients 1 and 2 undergoing intracoronary transplantation of allogeneic WJ-MSCs at baseline and follow-ups. LVEF: Left Ventricular Ejection Fraction, LV Tei Index or MPI: Myocardial performance index, TAPSE: Tricuspid Annular Plane Systolic Excursion, LVIDd: Left ventricular internal dimension at end-diastole. The variables of LVIDd and LVEDD are often used interchangeably in clinical practice. In this study, LVIDd is reported

During the three-month follow-up, the NYHA functional class improved from II/III to class II, and the NYU PHFI also decreased from 11 to 6 (Fig. 1). As indicators of improved quality of life (without the use of formal questionnaires), the patient’s appetite improved, resulting in a weight gain of at least 3 kg by the three-month follow-up. The patient was able to engage in swimming activities starting two weeks before the three-month follow-up. Additionally, the 6MWT showed an increasing trend from 300 to 373 m (Fig. 1). The NT-proBNP test showed a decreasing trend from 3502 to 1734 pg/ml (Fig. 1). On transthoracic echocardiography at the three-month follow-up, there was an improvement in both LVEF (from 33 to 48%) and TAPSE from 15 to 18 millimeters (mm). In addition, a decrease in left ventricular internal dimension during diastole (LVIDd) from 65 to 57 mm and the LV Tei index from 0.6 to 0.4% were observed (Fig. 2). As mentioned, prior to the scheduled time at the six-month follow-up, the patient was hospitalized due to a respiratory infection (COVID-19), and all variables showed a deteriorating trend. On CMR, the evaluation at baseline and at the six-month follow-up revealed a decreasing trend in LVEF (from 22 to 15%) and RVEF from 44 to 25%, with an increasing trend in LVEDV from 192 to 194 ml, LVESV from 149 to 165 ml, RVEDV from 100 to 113 ml, and RVESV from 55 to 85 ml, which confirmed the decompensated status of this patient (Fig. 1). Unfortunately, the patient experienced sudden cardiac death at 6 months of follow-up.

### Patient 2-demographic data

Patient 2 was a 7-year-old male weighing 20 kg. The disease was diagnosed at 5 months of age. The differential diagnosis of congenital dilated cardiomyopathy due to TORCH infections was ruled out. Drug history included ACEi (enalapril 2.5 mg once daily), aldosterone antagonists (spironolactone 12.5 mg once daily), diuretics (furosemide 10 mg once daily), and digoxin (Lanoxin syrup 50 µg twice daily). Baseline NYHA was II/III functional class. After obtaining informed consent from his parents, initial assessments showed an LVEF of 43% on echocardiography and 46% on CMR. Plasma NT-proBNP level was within the normal range at 20 pg/mL.

### Patient 2-efficacy outcomes (6MWT, NYHA, lab tests, echocardiography & CMR)

Over the 12-month follow-up, the NYHA functional class improved from II/III to class II, and the NYU PHFI had a decreasing trend from 9 to 5 (Fig. 1). Among indicators of quality of life, the patient’s appetite improved, along with the ability to participate in physical activities at school, and a weight gain of at least 1.5 kg by the three-month follow-up. The 6MWT also indicated an increasing trend from 265 to 380 m (Fig. 1). The plasma level of the NT-proBNP test had slight fluctuations; however, it remained in the normal range (Fig. 1). The echocardiographic findings showed an increasing trend in LVEF (from 43 to 64%) after remaining stable for the past three years. The minimal changes occurred in TAPSE (from 17 to 16.5 mm), LVIDd (from 58 to 42 mm), and the LV Tei index from 0.4 to 0.5% (Fig. 2). This patient experienced mild respiratory symptoms (a probable viral infection) at the 6-month follow-up, followed by transient fluctuations in clinical parameters. Additionally, CMR results at baseline, 6, and 12 months showed an increasing trend in LVEF (from 46 to 52%) and RVEF (from 56 to 63%), a slight increase in LVEDV (from 60 to 65 ml), and RVEDV (from 49 to 53 ml). Moreover, negligible changes in LVESV (from 32 to 31 ml) and RVESV (from 21 to 20 ml) were observed (Fig. 1).

### Safety and feasibility outcomes in patients 1 & 2

Intracoronary injections of allogeneic WJ-MSCs were feasible in both PDCM patients. There were undesirable consequences and major adverse cardiovascular events (MACE) during the follow-up period for patient 1. Following the intracoronary injection, non-specific ST-T changes and premature atrial contractions (PACs) were observed on the ECG during the first 24 h of serial heart monitoring. Troponin I was negative. To ensure safety, continuous monitoring was performed in the hospital for one week, after which the patient was discharged. The patient was admitted to the hospital 10 days before the scheduled follow-up appointment at month 6 due to a respiratory infection. COVID-19 infection was confirmed by 2 PCR tests. Unfortunately, she experienced sudden cardiac death at 6-month follow-up in subsequent decompensated heart failure. Her parents did not consent to an endomyocardial biopsy as the gold standard of diagnosis. No complications occurred in the second patient (Table 2).

**Table 2:** Adverse events and MACE

Adverse Events	Patient 1	Patient 2
Nonfatal stroke	**-**	**-**
Nonfatal myocardial infarction	**-**	**-**
Cardiovascular death	**+** (6 months follow-up)	**-**
Arrest / Dysrhythmia	**+** (PACs at first 24 h after cell transplantation)	**-**
Hospitalization for heart failure	**+** (Due to decompensation related to COVID19 Infection before 6 months follow-up)	**-**
Need to heart transplantation	**+** (Patient had already been listed for it prior to enrollment)	**-**

*MACE* Major Adverse Cardiovascular Events. +: event happend in the patient; –: event not happened in the patient

## Discussion

Cell therapy in pediatric patients with heart diseases focuses on two main categories: congenital heart disorders, such as hypoplastic left heart syndrome (HLHS), and cardiomyopathies, such as non-ischemic DCM. Supplementary Table 2 summarizes 12 published clinical studies on cell therapy for PDCM. The important point is that while all these previous studies focused on autologous cells, our study is the first to investigate allogeneic WJ-MSCs in PDCM, potentially offering an off-the-shelf therapeutic option that avoids the limitations of autologous cell sourcing.

Among these 12 studies, two involved ischemic cardiomyopathy [19, 20], while the remaining 10 focused on non-ischemic DCM [18, 21–29].

In terms of cell types, 10 studies used mononuclear cells (MNCs) or enriched subpopulations, such as CD34 + and CD133 + cells, derived from bone marrow or peripheral blood [19–28]. One study utilized BM-MSCs [18], and another used cardiosphere-derived cells (CDCs) [29]. The maximum dosages administered were 270 × 10⁶ for MNCs [21], 4.8 × 10⁶/ml administered in approximately 6 mL (total dose: 28.8 × 10⁶ cells) for MSCs [18], and 3.0 × 10⁵ for CDCs [29]. Delivery routes included intracoronary (9 studies) [18, 20–22, 24, 25, 27–29], intramyocardial (2 studies) [23, 26], and transcoronary transplantation (1 study) [19].

The dose of 1.5 × 10⁶ MSCs/kg used in this study was selected based on available evidence from MSC-based cardiac cell-therapy literature. Notably, two recent studies support dosing within this range. A meta-analysis of 11 randomized controlled trials including 1,098 participants reported that lower total MSC doses (< 100 million cells) were associated with more favorable improvements in left ventricular ejection fraction compared with higher doses (≥ 100 million cells) [30]. In addition, a recent systematic review of MSC therapy after myocardial infarction reported beneficial effects with intracoronary MSC doses in the range of approximately 1–3 × 10⁶ MSCs/kg [31], which is consistent with the dose used in the present study. However, the optimal dosing strategy for MSC therapy in pediatric dilated cardiomyopathy remains uncertain and requires confirmation in larger controlled studies.

The primary efficacy outcomes reported in most studies showed improvements in EF and NYHA class. However, one randomized trial reported no significant difference in the EF between the study groups [27].

Additionally, an ongoing trial (NCT06464588), categorized as a phase 1 open-label study, aims to assess the safety of repeated intravenous administration of allogeneic neonatal mesenchymal cells (nMSCs) in young adults (phase 1 A) and pediatric patients (phase 1B) with DCM. This trial plans to administer escalating dose levels at 0, 15, and 30 days.

### Safety aspects

The novelty of the present “A HEART-WISE” study lies in the application of allogeneic MSCs as an off-the-shelf therapeutic approach in pediatric dilated cardiomyopathy (PDCM). In this preliminary experience, intracoronary administration of allogeneic WJ-MSCs was technically feasible, and no procedure-related major adverse cardiac events were observed. However, given the very small sample size and the observational nature of this report, these findings should be interpreted with caution and cannot be considered definitive evidence of safety. Patient 1’s death, which occurred in the context of COVID-19 infection and was unrelated to the intervention, highlights the vulnerability of patients with advanced cardiomyopathy to systemic complications. In patient 2, the absence of major complications during follow-up is consistent with prior reports suggesting a favorable safety profile of allogeneic MSC therapy in cardiomyopathy [8, 14]. Larger controlled studies are required to more reliably establish the safety of intracoronary MSC administration in pediatric patients.

### Efficacy aspects

Although there have been over two decades of experience with cardiac cell therapy, a universal consensus on standardized endpoints has yet to be established. This is due to the numerous influencing factors associated with these new therapeutic approaches, such as the nature of the disease, cell types, cell preparation methods, cell doses, routes of administration, timing of the intervention, and the frequency of repeating treatments [32, 33].

Banovic et al. suggested surrogate endpoints for ischemic heart failure trials, focusing on various categories including symptoms, functional outcomes, paraclinical assessments, and quality of life. They also proposed a threshold for clinical significance for these endpoints [32]. Studies have shown improvements in LVEF for both ischemic and non-ischemic cardiomyopathy patients who have received stem cell-based treatment [34, 35]. While LVEF is commonly used as a primary endpoint, several confounding factors can limit its reliability as the sole measure of therapeutic efficacy. Accordingly, Dorobantu et al. suggested that quality of life and performance status analyses should receive increasing attention, as they offer complementary insights into the patient’s perspective of treatment outcomes [33].

In this study, clinically significant improvements in both patients were defined as at least a one-class reduction in NYHA, a more than 50-meter increase in the 6MWT, and a 5% increase in LVEF, as assessed by echocardiography and CMR. A declining pattern in LVIDd was observed. Improvements in performance status were also reflected in quality-of-life indicators, as described in the results section. A reduction in NT-proBNP levels by 1768 pg/ml (greater than 300 pg/ml) in patient 1 indicates clinical evidence of improved cardiac function. The NT-proBNP levels of patient 2 were in normal range of lab test reports even before intervention. Due to the viral infection and subsequent heart failure decompensation in patient 1, these data apply to the first patient at the 3-month and the second patient at the 1-year follow-up duration.

### Limitations and future directions

The small sample size limits generalizability and statistical analysis of therapeutic efficacy. Viral infections in both patients during the follow-up period highlight the importance of managing external factors, such as intervening infections, that may impact trial results. Future efforts should explore Cell-derived extracellular vesicles (EVs) for reducing immunogenic and infection-related risks [29], particularly as a potential alternative for maintaining therapeutic effects in clinically unstable patients. EVs may offer a simpler route of administration during the transition until recovery to stable conditions.

A discrepancy between echocardiographic and CMR‑derived LVEF values was observed in our patients. Such variability between imaging modalities is well recognized, particularly in dilated cardiomyopathy. Echocardiographic LVEF estimation using the biplane Simpson method may be affected by image quality, geometric assumptions, and potential foreshortening of apical views or suboptimal endocardial border delineation [36]. In contrast, CMR provides three‑dimensional volumetric assessment using a complete short‑axis stack and is considered the reference standard for quantifying ventricular volumes and ejection fraction [37, 38].

In the context of our study, several additional factors should also be considered. Echocardiography and CMR examinations were performed at different centers and not always on the same day. The interval between the two assessments was approximately two weeks in patient 1 and two days in patient 2. Temporal differences between examinations, together with inherent methodological differences between imaging modalities, may therefore explain the observed variation in LVEF measurements.

Myocardial strain analysis, such as global longitudinal strain, may provide additional insights into myocardial function. However, strain imaging was not routinely incorporated into the echocardiographic protocol at our center during the period when these patients were evaluated, and dedicated acquisitions for speckle‑tracking analysis were not systematically obtained. Consequently, strain measurements could not be reliably assessed retrospectively from the available echocardiographic datasets. CMR, which provides highly reproducible and less operator‑dependent assessment of ventricular function, was used to complement echocardiographic evaluation.

Another limitation of the present report is that immunological monitoring, including assessment of immune cell subsets (e.g., regulatory T cells, NK cells, and T lymphocytes) or inflammatory cytokines, was not performed. Such analyses could provide additional insight into the host immune response following administration of allogeneic mesenchymal stromal cells. However, MSCs are generally considered to have relatively low immunogenicity, characterized by low expression of HLA class I molecules, absence of HLA class II expression under basal conditions, and lack of key costimulatory molecules involved in T-cell activation [39, 40]. Consistent with these biological properties, several clinical studies have demonstrated the safety of allogeneic MSC therapy without routine immunosuppressive treatment. For example, the POSEIDON trial reported no significant donor-specific alloimmune reactions after transendocardial administration of allogeneic MSCs [14], and the CAREMI trial evaluating intracoronary MSC therapy similarly reported no immune-related adverse events [41]. Larger systematic analyses have likewise found no increased risk of immune rejection or infection associated with MSC therapy [42, 43]. Despite these findings, further studies incorporating systematic immunological monitoring and longer follow-up are warranted to better characterize the immune response to allogeneic MSC therapy.

Excluding the unfavorable outcome of patient 1, clinical symptoms and paraclinical variables indicated significant cardiac improvement prior to the onset of the viral infection. Given her history of chemotherapy-induced DCM, this observation may support the exploration of new therapeutic or preventive strategies for this subtype of DCM, in continuity with previous studies [8, 44, 45] and an ongoing trial [NCT02962661].

Finally, several methodological considerations should be acknowledged when interpreting the present findings. The very small sample size does not allow definitive conclusions regarding the safety of intracoronary administration of allogeneic MSCs in pediatric dilated cardiomyopathy. Although no major adverse cardiac events or severe complications were observed in these two patients, these observations should be interpreted cautiously and cannot be considered sufficient evidence of safety. Larger, well-designed controlled studies are required to further evaluate the safety of this therapeutic approach.

In addition, both patients were receiving ongoing medical therapy consistent with current treatment approaches for pediatric dilated cardiomyopathy. Therefore, the potential contribution of background pharmacological therapy to the observed clinical improvement cannot be completely excluded. However, both patients had received similar medical regimens for prolonged periods prior to MSC administration, more than one year in patient 1 and more than three years in patient 2,without meaningful clinical improvement. During follow-up after MSC therapy, background treatment remained largely unchanged, with only minor adjustments in digoxin dosage or formulation and no modification of the overall therapeutic strategy.

Although a temporal association was observed between MSC administration and subsequent improvement in cardiac function, the small sample size and observational nature of this case reports prevent definitive conclusions regarding causality. Larger controlled studies will be necessary to clarify the independent contribution of cell-based therapy beyond standard medical treatment.

## Conclusion

This study provides preliminary evidence supporting the feasibility and potential therapeutic role of WJ-MSCs in pediatric DCM. Larger, well-controlled trials are needed to confirm these observations and optimize cell-based therapies, including the ideal timing and dosage.

## Supplementary Information

Supplementary Material 1.

Supplementary Material 2.

## Data Availability

The datasets generated and/or analyzed during the current study, including clinical findings, imaging data, and laboratory test results, are not publicly available in order to protect patient privacy as per ethical guidelines. However, the data are available from the corresponding author on reasonable request, provided the request complies with institutional and ethical guidelines for data sharing.

## Abbreviations

6MWT: Six-minute walk test
Al: Allogeneic
At: Autologous
BM-MNC: Bone marrow-derived mononuclear cells
BM-MSC: Bone marrow-derived mesenchymal stromal cells
CDC: Cardiosphere-derived cells
CMR: Cardiac magnetic resonance
COA: Certificate of analysis
CRF: Case report form
DCM (i): ischemic DCM
DCM: Dilated cardiomyopathy
EVs: Extracellular vesicles
F/U: Follow-up
F: Female
HTX: Heart transplantation
IC: Intracoronary
IM: Intramyocardial
LFT: Liver function tests
LVEDD: Left ventricular end diastolic diameter
LVEDV: Left ventricular end diastolic volume
LVEF: Left ventricular ejection fraction
LVESD: Left ventricular end systolic diameter
LVESV: Left ventricular end systolic volume
LVIDd: Left ventricular internal dimension at end-diastole
M: Male
m: Months
MACE: Major adverse cardiovascular events
MPI: Myocardial performance index
MSCs: Mesenchymal stromal cells
NA: Not applicable
NIDCM: Non-ischemic dilated cardiomyopathy
NT-proBNP: N-terminal pro-brain natriuretic peptide
NYHA: New York heart association
NYU PHFI: New York university pediatric heart failure index
PACs: Premature atrial contractions
PB-MNC: Peripheral blood mononuclear cells
PDCM: Pediatric dilated cardiomyopathy
RVEDV: Right ventricular end diastolic volume
RVEF: Right ventricular ejection fraction
RVESV: Right ventricular end systolic volume
TAPSE: Tricuspid annular plane systolic excursion
TC: Transcoronary
ULN: Upper limits of normal
W: Week
WJ-MSCs: Wharton’s jelly-derived MSCs
Y: Years old
