# Supplementary material for: A HEART-WISE: Allogeneic Wharton’s Jelly-derived Mesenchymal Stromal Cells’ Intracoronary Transplantation in Pediatric Patients with Dilated Cardiomyopathy: The First Case Reports
Source: BMC Cardiovasc Disord. 2026 Apr 27;26:509. doi: 10.1186/s12872-026-05866-x (PMC13270590; doi:10.1186/s12872-026-05866-x)
Supplement: Supplementary file 1 — Supplementary Material 1. [file 12872_2026_5866_MOESM1_ESM.pdf]

Case Report Form

Title

The Safety & Feasibility of Wharton's Jelly Derived-Mesenchymal Stem Cells Transplantation in Pediatric with Non-Ischemic Dilated Cardiomyopathy (WJ-MSC PNIDCM): Clinical Trial Phase I

Sponsor

Cell Tech Pharmed
Address: Celltechphamed Co., Tolid Darou Pharmaceutical Complex, Kermany st., Moallem Blv., Yaftabad, Tehran, Iran.
Tel: +98-21-67357000
Fax: +98-21-67357200
P.O. Box: 1371616314, Tehran, Iran
Email: info@celltech.co
http://celltech.ir/

Principal Investigator (s)

Dr. Aliakbar Zeinaloo
Podiatrists - MD
Professor of paediatric cardiology, Children's Hospital Medical Center, Tehran University of Medical Sciences
Tel: +98-21-88024475
Email: zeinaloo@tums.ac.ir

Dr. Nasser Aghdami
Medical Immunology PhD - MD
Associate Professor of Royan Institute
Tel: +98-21-22518388
Email: nasser.aghdami@royaninstitute.org

Time flow

Time	-3W	0	24h	1 W	1 M	3 M	6 M	9 M	12 M
Visit #	0	1	2	3	4	5	6	7	8
Consent	■								
Eligibility assessment	■								
Ph.Ex	■		■	■	■	■	■	■	■
EKG	■		■	■	■	■	■	■	■
Echocardiography	■		■	■	■	■	■	■	■
P NYHA	■		■	■	■	■	■	■	■
Lab test 1	■								■
Lab test 2	■								
Lab test 3	■			■	■	■	■	■	■
6MWT	■			■	■	■	■	■	■
CMR	■						■		■
Intervention ± EMB		■							
Adverse events		■	■	■	■	■	■	■	■

WJ-MSC Site No. |___|___| **ID No.** |___|___| **Patient's Initials:** |___|___|___|___|

Table: Lab Tests details

Lab Tests 1	Lab Test 2	Lab Test 3
CBC, diff<br>ESR, CRP	HBs Ag, HBs Ab, HBC Ab, HCV Ab, HIV Ab, CMV Ab	Troponin I & Pro BNP
BUN, Cr<br>Na, K, Ca, Mg, p<br>Uric acid		
FBS/BS, HbA1C (if needed)		
(25) OH vit D3 (if needed)		
PT, PTT, INR		
U/A, U/C		
TSH, T3, T4		
AST, ALT, ALP, Bill T&D		
Serum Iron, TIBC, Ferritin		
CPK, LDH, CKMB		
Chol, LDL, HDL, TG (if needed)		

Tick here if page is void: |___|
Investigator's signature: ………………… **Date** (dd/mm/yy): |___|___|/|___|___|/|___|___|

Visit 0 (-3 W)

Date (dd/mm/yy): |___|___|/|___|___|/|___|___|

Informed consent? **Yes:** □ **No:** □

Date of birth (dd/mm/yyyy):	|__|__|/|__|__|/|__|__|__|__| Age |___|___| Sex: F: □ M: □

History Taking
*** Please describe:**

Physical examination:
*** Please describe:**

MD Name:

Visit 0 (-3 W)

Date (dd/mm/yy): |___|___|/|___|___|/|___|___|

Inclusion criteria*	Yes	No *
• Age: 4-18 y	☐	☐
• Both gender	☐	☐
• Chronic heart failure disease & progressive symptoms (more than 6 months)	☐	☐
• No response to standard therapy (more than 3 months)	☐	☐
• NYHA function class II/III (NYH PHFI)	☐	☐
• 20<LVEF (echo)%<45 (echocardiography)	☐	☐
• Informed consent	☐	☐
* If a single "No" is ticked, the volunteer is not eligible for the trial		

Exclusion criteria*	Yes	No *
• Secondary cause for reduced EF<br>○ Including critical valvular disease, severe coarctation, coronary artery anomalies, metabolic disorders, neuromuscular disorders, congenital cardiac disorders except mitral valve prolapse (MVP)	☐	☐
• Active malignancy	☐	☐
• Cardiogenic/ toxic shock	☐	☐
• Active infectious disease/ Positive viral markers (HIV, HBV, HCV, …)	☐	☐
• Immunodeficiency diseases	☐	☐
• Arrhythmia	☐	☐
• Uncontrolled underlying disease<br>o LFT ≥ 3 ULN<br>o Cr > 2 mg/dl	☐	☐
• * If a single "Yes" is ticked, the volunteer is not eligible for the trial		

Tick here if page is void: |___|

Investigator's signature: ………………… **Date** (dd/mm/yy): |___|___|/|___|___|/|___|___|

**Is the volunteer eligible for the trial?**	**Yes: □**	**No: □**
**Patient's center and ID number*:** |___|___|___|___| **Patient's initials**:** |___|___|___|___|		

*** First 2 numbers are the site numbers; second 2 numbers are patient's file number.**
****First 2 letters of first name, first 2 letters of last name.**

	Yes	No	Comment/Score
Ph.Ex	☐	☐	
EKG	☐	☐	
Echocardiography	☐	☐	
NYHA	☐	☐	
6MWT	☐	☐	
Lab Test 1	☐	☐	
Lab Test 2	☐	☐	
Lab Test 3	☐	☐	
CMR	☐	☐	
Any consideration (if):			

WJ-MSC Site No. |___|___| **ID No.** |___|___| **Patient's Initials:** |___|___|___|___|

Visit 1 (0 day)

Date (dd/mm/yy): |___|___|/|___|___|/|___|___|

	Yes	No	Comment/Score
Intervention ± EMB	☐	☐	
Cell Count	☐	☐	
Cell Viability	☐	☐	
Any Description:			

Visit 1 (0 day)

Date (dd/mm/yy): |___|___|/|___|___|/|___|___|

**Description of Events** (According to appendix – in protocol)	**Onset Date** dd/ mm/	**Ended Date** dd/ mm/	**Intensity** (Record maximum intensity during occurrence period) Mild = 1 Moderate = 2 Severe = 3	**Causality** Not related = 0 Unlikely = 1 Possible = 2 Probable = 3 Most probable = 4 Insufficient data to assess = 5	**Seriousness** Non-serious = 0 Serious = 1 (If serious, complete a SAE form)	**Action taken** No = 0 Yes = 1* * If a treatment is given, please complete form
	|__|__| / |__|__|	|__|__|/ |__|__|	|__|	|__|	|__|	|__|
	|__|__| / |__|__|	|__|__|/ |__|__|	|__|	|__|	|__|	|__|
	|__|__| / |__|__|	|__|__|/ |__|__|	|__|	|__|	|__|	|__|

Tick here if page is void: |___|

Investigator's signature: ………………… **Date** (dd/mm/yy): |___|___|/|___|___|/|___|___|

Visit 2 (24 h)

Date (dd/mm/yy): |___|___|/|___|___|/|___|___|

	Yes	No	Comment/Score
Ph.Ex	☐	☐	
EKG	☐	☐	
Echocardiography	☐	☐	
NYHA	☐	☐	
Discharge	☐	☐	
Any Description:			

Visit 2 (24 h)

Date (dd/mm/yy): |___|___|/|___|___|/|___|___|

**Description of Events** (According to appendix – in protocol)	**Onset Date** dd/ mm/	**Ended Date** dd/ mm/	**Intensity** (Record maximum intensity during occurrence period) Mild = 1 Moderate = 2 Severe = 3	**Causality** Not related = 0 Unlikely = 1 Possible = 2 Probable = 3 Most probable = 4 Insufficient data to assess = 5	**Seriousness** Non-serious = 0 Serious = 1 (If serious, complete a SAE form)	**Action taken** No = 0 Yes = 1* * If a treatment is given, please complete form
	|__|__| / |__|__|	|__|__|/ |__|__|	|__|	|__|	|__|	|__|
	|__|__| / |__|__|	|__|__|/ |__|__|	|__|	|__|	|__|	|__|
	|__|__| / |__|__|	|__|__|/ |__|__|	|__|	|__|	|__|	|__|

Visit 3 (1 W)<br>Date (dd/mm/yy): |___|___|/|___|___|/|___|___|			
	**Yes**	**No**	**Report**
Ph.Ex	☐	☐	
EKG	☐	☐	
Echocardiography	☐	☐	
NYHA	☐	☐	
6MWT	☐	☐	
Lab Test 3	☐	☐	
Any Description:			

Visit 3 (1 W)<br>Date (dd/mm/yy): |___|___|/|___|___|/|___|___|						
**Description of Events**<br>(According to appendix – in protocol)	**Onset Date**<br>dd/<br>mm/	**Ended Date**<br>dd/<br>mm/	**Intensity**<br>(Record maximum intensity during occurrence period)<br>Mild = 1<br>Moderate = 2<br>Severe = 3	**Causality**<br>Not related = 0<br>Unlikely = 1<br>Possible = 2<br>Probable = 3<br>Most probable = 4<br>Insufficient data to assess = 5	**Seriousness**<br>Non-serious = 0<br>Serious = 1<br>(If serious, complete a SAE form)	**Action taken**<br>No = 0<br>Yes = 1*<br>* If a treatment is given , please complete form
	|__|__| /<br>|__|__|	|__|__| /<br>|__|__|	|__|	|__|	|__|	|__|
	|__|__| /<br>|__|__|	|__|__| /<br>|__|__|	|__|	|__|	|__|	|__|

Tick here if page is void: |___|
Investigator's signature: ………………… **Date** (dd/mm/yy): |___|___|/|___|___|/|___|___|

WJ-MSC Site No. |___|___| **ID No.** |___|___| **Patient's Initials:** |___|___|___|___|

Visit 4 (1 M)

Date (dd/mm/yy): |___|___|/|___|___|/|___|___|

	Yes	No	Report
Ph.Ex	☐	☐	
EKG	☐	☐	
Echocardiography	☐	☐	
NYHA	☐	☐	
6MWT	☐	☐	
Lab Test 3	☐	☐	

Any Description:

Visit 4 (1 M)

Date (dd/mm/yy): |___|___|/|___|___|/|___|___|

**Description of Events** (According to appendix – in protocol)	**Onset Date** dd/ mm/	**Ended Date** dd/ mm/	**Intensity** (Record maximum intensity during occurrence period) Mild = 1 Moderate = 2 Severe = 3	**Causality** Not related = 0 Unlikely = 1 Possible = 2 Probable = 3 Most probable = 4 Insufficient data to assess = 5	**Seriousness** Non-serious = 0 Serious = 1 (If serious, complete a SAE form)	**Action taken** No = 0 Yes = 1* * If a treatment is given , please complete form
	|__|__| / |__|__|	|__|__| / |__|__|	|__|	|__|	|__|	|__|
	|__|__| / |__|__|	|__|__| / |__|__|	|__|	|__|	|__|	|__|

Tick here if page is void: |___|
Investigator's signature: ………………… **Date** (dd/mm/yy): |___|___|/|___|___|/|___|___|

WJ-MSC Site No. |___|___| **ID No.** |___|___| **Patient's Initials:** |___|___|___|___|

Visit 5 (3 M)

Date (dd/mm/yy): |___|___|/|___|___|/|___|___|

	Yes	No	Report
Ph.Ex	☐	☐	
EKG	☐	☐	
Echocardiography	☐	☐	
NYHA	☐	☐	
6MWT	☐	☐	
Lab Test 3	☐	☐	

Any Description:

Visit 5 (3 M)

Date (dd/mm/yy): |___|___|/|___|___|/|___|___|

**Description of Events** (According to appendix – in protocol)	**Onset Date** dd/ mm/	**Ended Date** dd/ mm/	**Intensity** (Record maximum intensity during occurrence period) Mild = 1 Moderate = 2 Severe = 3	**Causality** Not related = 0 Unlikely = 1 Possible = 2 Probable = 3 Most probable = 4 Insufficient data to assess = 5	**Seriousness** Non-serious = 0 Serious = 1 (If serious, complete a SAE form)	**Action taken** No = 0 Yes = 1* * If a treatment is given , please complete form
	|__|__| / |__|__|	|__|__| / |__|__|	|__|	|__|	|__|	|__|
	|__|__| / |__|__|	|__|__| / |__|__|	|__|	|__|	|__|	|__|

Tick here if page is void: |___|

Investigator's signature: ………………… **Date** (dd/mm/yy): |___|___|/|___|___|/|___|___|

WJ-MSC Site No. |___|___| **ID No.** |___|___| **Patient's Initials:** |___|___|___|___|

**Visit 6 (6 M)**<br>**Date** (dd/mm/yy): |___|___|/|___|___|/|___|___|			
	**Yes**	**No**	**Report**
Ph.Ex	☐	☐	
EKG	☐	☐	
Echocardiography	☐	☐	
NYHA	☐	☐	
6MWT	☐	☐	
Lab Test 3	☐	☐	
CMR	☐	☐	
Any Description:			

**Visit 6 (6 M)**<br>**Date** (dd/mm/yy): |___|___|/|___|___|/|___|___|						
**Description of Events** (According to appendix – in protocol)	**Onset Date** dd/ mm/	**Ended Date** dd/ mm/	**Intensity** (Record maximum intensity during occurrence period) Mild = 1 Moderate = 2 Severe = 3	**Causality** Not related = 0 Unlikely = 1 Possible = 2 Probable = 3 Most probable = 4 Insufficient data to assess = 5	**Seriousness** Non-serious = 0 Serious = 1 (If serious, complete a SAE form)	**Action taken** No = 0 Yes = 1* * If a treatment is given , please complete form
	|__|__| / |__|__|	|__|__| / |__|__|	|__|	|__|	|__|	|__|
	|__|__| / |__|__|	|__|__| / |__|__|	|__|	|__|	|__|	|__|

Tick here if page is void: |___|
Investigator's signature: ………………… **Date** (dd/mm/yy): |___|___|/|___|___|/|___|___|

WJ-MSC Site No. |___|___| **ID No.** |___|___| **Patient's Initials:** |___|___|___|___|

Visit 7 (9 M)

Date (dd/mm/yy): |___|___|/|___|___|/|___|___|

	Yes	No	Report
Ph.Ex	☐	☐	
EKG	☐	☐	
Echocardiography	☐	☐	
NYHA	☐	☐	
6MWT	☐	☐	
Lab Test 3	☐	☐	

Any Description:

Visit 7 (9 M)

Date (dd/mm/yy): |___|___|/|___|___|/|___|___|

**Description of Events** (According to appendix – in protocol)	**Onset Date** dd/ mm/	**Ended Date** dd/ mm/	**Intensity** (Record maximum intensity during occurrence period) Mild = 1 Moderate = 2 Severe = 3	**Causality** Not related = 0 Unlikely = 1 Possible = 2 Probable = 3 Most probable = 4 Insufficient data to assess = 5	**Seriousness** Non-serious = 0 Serious = 1 (If serious, complete a SAE form)	**Action taken** No = 0 Yes = 1* * If a treatment is given , please complete form
	|__|__| / |__|__|	|__|__| / |__|__|	|__|	|__|	|__|	|__|
	|__|__| / |__|__|	|__|__| / |__|__|	|__|	|__|	|__|	|__|

Tick here if page is void: |___|

Investigator's signature: ………………… **Date** (dd/mm/yy): |___|___|/|___|___|/|___|___|

Visit 8 (12 M)

Date (dd/mm/yy): |___|___|/|___|___|/|___|___|

	Yes	No	Report
Ph.Ex	☐	☐	
EKG	☐	☐	
Echocardiography	☐	☐	
NYHA	☐	☐	
6MWT	☐	☐	
Lab Test 3	☐	☐	
Lab Test 1	☐	☐	
CMR	☐	☐	

Any Description:

Visit 8 (12 M)

Date (dd/mm/yy): |___|___|/|___|___|/|___|___|

**Description of Events** (According to appendix – in protocol)	**Onset Date** dd/ mm/	**Ended Date** dd/ mm/	**Intensity** (Record maximum intensity during occurrence period) Mild = 1 Moderate = 2 Severe = 3	**Causality** Not related = 0 Unlikely = 1 Possible = 2 Probable = 3 Most probable = 4 Insufficient data to assess = 5	**Seriousness** Non-serious = 0 Serious = 1 (If serious, complete a SAE form)	**Action taken** No = 0 Yes = 1* * If a treatment is given , please complete form
	|__|__| / |__|__|	|__|__| / |__|__|	|__|	|__|	|__|	|__|
	|__|__| / |__|__|	|__|__| / |__|__|	|__|	|__|	|__|	|__|

Tick here if page is void: |___|

Investigator's signature: ………………… **Date** (dd/mm/yy): |___|___|/|___|___|/|___|___|

Trial Completion

**Did participant complete the trial?**	☐ **Yes,** please provide **date of last visit**: __ __ / __ __ / 2 0 __ __ (DD / MM / YYYY)<br>☐ **No,** please provide **date of withdrawal** and complete below: __ __ / __ __ / 2 0 __ __ (DD / MM / YYYY)

Early Withdrawal: please tick most appropriate reason for participant not completing the trial:

☐ **Adverse Events related:** please state related AE: ______________________

☐ **Participant's decision, specify: ______________________________**

☐ **Investigator's decision, specify: ______________________________**

☐ **Sponsor's decision, specify: ______________________________**

☐ **Lost to follow up, specify: ______________________________**

☐ **Other, specify: __________________**

Please indicate the reason in the case of drop-out:

	Criteria		Explanation
1	The patient became pregnant.	YES ☐ NO☐	
2	The patient could not tolerate intervention	YES ☐ NO☐	
3	The patient died.	YES ☐ NO☐	
4	The patient suffered from life-threatening complications due to intervention	YES ☐ NO☐	
5	The patient suffered from life-threatening complications due to other cause (indicate it in details)	YES ☐ NO☐	
7	The patient suffered from any disease or other medical conditions that make it difficult to continue the study:<br>YES ☐ NO☐<br>Note it:		

Tick here if page is void: |___|

Investigator's signature: ………………… **Date** (dd/mm/yy): |___|___|/|___|___|/|___|___|

Principal Investigator's Sign Off

**Principal Investigator's Signature Statement:**	
I have reviewed this CRF and confirm that, to the best of my knowledge, it accurately reflects the study information obtained for this participant. All entries were made either by myself or by a person under my supervision who has signed the Delegation and Signature Log.	
**Principal Investigator's Name:**	**Date of Signature:** _ __/ __ __/ __ __ __ __<br>(DD / MM / YYYY)
**Principal Investigator's Signature:**	
**ONCE SIGNED, NO FURTHER CHANGES CAN BE MADE TO THIS CRF WITHOUT A SIGNED DATA QUERY FORM.**	
