# Supplementary material for: A HEART-WISE: Allogeneic Wharton’s Jelly-derived Mesenchymal Stromal Cells’ Intracoronary Transplantation in Pediatric Patients with Dilated Cardiomyopathy: The First Case Reports
Source: BMC Cardiovasc Disord. 2026 Apr 27;26:509. doi: 10.1186/s12872-026-05866-x (PMC13270590; doi:10.1186/s12872-026-05866-x)
Supplement: Supplementary file 2 — Supplementary Material 2. [file 12872_2026_5866_MOESM2_ESM.docx]

Supplementary Figure 1- Flow Cytometry Analysis
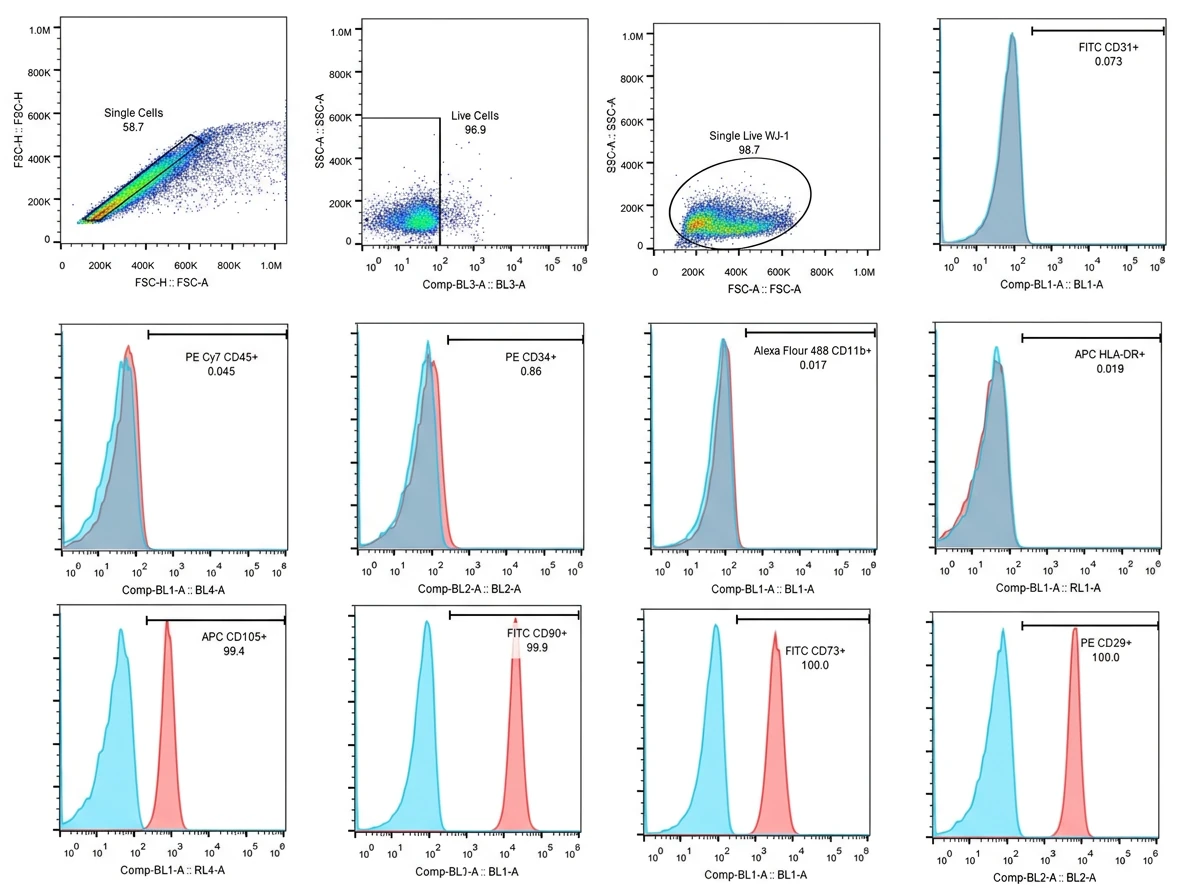


Supplementary Figure 1. Flow cytometric analysis of WJ-MSCs performed at the working cell bank level (passage 3) to confirm ≥95% expression of CD73, CD90, CD105, and CD29, and ≤2% expression of CD45, CD34, CD31, CD11b, and HLA‑DR, in accordance with ISCT criteria.

Supplementary Table 1- Quality Control Tests

| Factors (accepted value) | P1 | P2 |
| --- | --- | --- |
| Cell count (1.5 × 10^6^ cells/kg) | 70 ×10^6^ | 30 ×10^6^ |
| Cell Viability (≥80%) | 93.6 | 94.2 |
| Sterility | Passed | Passed |
| Mycoplasma | Not detected | Not detected |
| Bacterial endotoxin (<0.25 EU/ml) | Negative | Negative |
| Expression of positive markers (≥95%) *  Including  CD73  CD90  CD105  CD29 | ≥95%  100%  99.9%  99.4%  100% | ≥95%  100%  99.9%  99.4%  100% |
| Expression of negative markers (≤2%) *  including  CD45  CD34  CD31  CD11b  HLA-DR | ≤1%  0.045%  0.86%  0.073%  0.017%  0.019% | ≤1%  0.045%  0.86%  0.073%  0.017%  0.019% |
| Karyotype | Normal | Normal |

Supplementary Table 1. Quality control test results of GMP-grade WJ-MSCs administered to each patient. *Flow cytometric immunophenotyping was performed at the working cell bank level (passage 3), while all other quality control assessments were conducted separately for each patient.

Supplementary Table 2- Published clinical cell therapy studies in pediatric with cardiomyopathy

| Main results | F/U | Route | Dose | Cell | Sex | Age | Size | Type | Ref. | No. |
| --- | --- | --- | --- | --- | --- | --- | --- | --- | --- | --- |
| EF (↑)  BNP (↓)  NYHA (↓) | 6 m | IC | 270×10^6^ | (At)  BM-MNC | M | 2y | 1 | DCM | 2009  Rupp et al,  [[21](file:///Y:\Repos\Gueen-neeeeeeever%20e%20delete\OUTPUT\2026\APR\18_CE\12872_2026_5866_Article\SOURCE\Manuscript_final_NN_V4_clean%20version.docx#_ENREF_21)] | 1. |
| EF (↑)  NYHA (↓) | 2 m  &  6 m | IC | 1.96× 10^6^/kg  1.27× 10^6^/kg | (At)  PB-MNC | M &  F | 6y  &  9y | 2 | DCM | 2010  Olguntürk et al,  [[22](file:///Y:\Repos\Gueen-neeeeeeever%20e%20delete\OUTPUT\2026\APR\18_CE\12872_2026_5866_Article\SOURCE\Manuscript_final_NN_V4_clean%20version.docx#_ENREF_22)] | 2. |
| EF (↑)  NYHA (↓) | 3 m | TC | NA  (20ml) | (At)  BM-MNC | F | 9y | 1 | DCM  (i) | 2010  Limsuwan et al,  [[19](file:///Y:\Repos\Gueen-neeeeeeever%20e%20delete\OUTPUT\2026\APR\18_CE\12872_2026_5866_Article\SOURCE\Manuscript_final_NN_V4_clean%20version.docx#_ENREF_19)] | 3. |
| EF (↑)  LVEDD (↓) | 4 m | IM | 20×10^6^ | (At)  BM-MNC | F | 3.5 m | 1 | DCM | 2011  Lacis et al,  [[23](file:///Y:\Repos\Gueen-neeeeeeever%20e%20delete\OUTPUT\2026\APR\18_CE\12872_2026_5866_Article\SOURCE\Manuscript_final_NN_V4_clean%20version.docx#_ENREF_23)] | 4. |
| EF (↑) | 1 y | IC | 4.8×10^6^/ml | (At)  BM-MSC | M | 11y | 1 | DCM | 2011  Zeinaloo et al,  [[18](file:///Y:\Repos\Gueen-neeeeeeever%20e%20delete\OUTPUT\2026\APR\18_CE\12872_2026_5866_Article\SOURCE\Manuscript_final_NN_V4_clean%20version.docx#_ENREF_18)] | 5. |
| EF (↑)  BNP (↓) | 4 m | IC | 6.15×10^6^/kg  10.55×10^6^/kg | (At)  PB-MNC | M | 3m  &  4m | 2 | DCM | 2011  Rivas et al,  [[24](file:///Y:\Repos\Gueen-neeeeeeever%20e%20delete\OUTPUT\2026\APR\18_CE\12872_2026_5866_Article\SOURCE\Manuscript_final_NN_V4_clean%20version.docx#_ENREF_24)] | 6. |
| 3 patients HTX,  1 patient died, others:  EF (↑)  BNP (↓)  NYHA (↓) | 1-52 m | IC | 5.5× 10^6^ | (At)  BM-MNC | NA | 4m to  16y | 9 | DCM | 2012  Rupp et al,  [[25](file:///Y:\Repos\Gueen-neeeeeeever%20e%20delete\OUTPUT\2026\APR\18_CE\12872_2026_5866_Article\SOURCE\Manuscript_final_NN_V4_clean%20version.docx#_ENREF_25)] | 7. |
| EF (↑)  BNP (↓)  NYHA (↓) | 1y | IM | 17-22× 10^6^ | (At)  BM-MNC | NA | 4m to  17y | 7 | DCM | 2013  Bergmane et al,  [[26](file:///Y:\Repos\Gueen-neeeeeeever%20e%20delete\OUTPUT\2026\APR\18_CE\12872_2026_5866_Article\SOURCE\Manuscript_final_NN_V4_clean%20version.docx#_ENREF_26)] | 8. |
| Cell/Placebo group comparison:  EF (↔)  EDV (↓) in cell  ESV (↓) in cell | 6m | IC | 11.8-115.1×10^6^ | (At)  BM-MNC | 6M  &  4F | 2 to 14 y | 10  (5/5) | DCM | 2017  Pincott et al,  [[27](file:///Y:\Repos\Gueen-neeeeeeever%20e%20delete\OUTPUT\2026\APR\18_CE\12872_2026_5866_Article\SOURCE\Manuscript_final_NN_V4_clean%20version.docx#_ENREF_27)] | 9. |
| EF (↑)  NYHA (↓) | 6m | IC | 21-73× 10^6^ | (At)  BM-MNC | 3M  &  5F | 4 to 15y | 8 | DCM | 2019  Amoozgar et al,  [[28](file:///Y:\Repos\Gueen-neeeeeeever%20e%20delete\OUTPUT\2026\APR\18_CE\12872_2026_5866_Article\SOURCE\Manuscript_final_NN_V4_clean%20version.docx#_ENREF_28)] | 10. |
| EF (↑)  BNP (↓)  NYHA (↓) | 4m | IC | 84 × 10^6^ | (At)  BM-MNC | M | 18  days | 1 | DCM  (i) | 2019  Zschirnt et al,  [[20](file:///Y:\Repos\Gueen-neeeeeeever%20e%20delete\OUTPUT\2026\APR\18_CE\12872_2026_5866_Article\SOURCE\Manuscript_final_NN_V4_clean%20version.docx#_ENREF_20)] | 11. |
| EF (↑)  BNP (↓)  ROSS (↓) | 6m | IC | 3.0×10^5^ | (At)  CDC | NA | 1-17y | 5 | DCM | 2020  Hirai et al,  [[29](file:///Y:\Repos\Gueen-neeeeeeever%20e%20delete\OUTPUT\2026\APR\18_CE\12872_2026_5866_Article\SOURCE\Manuscript_final_NN_V4_clean%20version.docx#_ENREF_29)] | 12. |

Al: Allogeneic, At: Autologous, BM-MNC: Bone marrow derived mono-nuclear cells, BM-MSC: Bone marrow derived mesenchymal stromal cells, BNP: Brain natriuretic peptide, CDC: Cardiosphere-derived cells, DCM: Dilated cardiomyopathy, DCM (i): ischemic DCM, EDV: End diastolic volume, EF: Ejection fraction, ESV: End systolic volume, F: Female, F/U: Follow up, HTX: Heart transplantation, IC: Intracoronary, IM: Intramyocardial, M: Male, m: Months, NA: Not applicable, NYHA: New York heart association, PB-MNC: Peripheral blood mono-nuclear cells, TC: Transcoronary, W: Week, Y: Years old
